# A 6 lncRNA-Based Risk Score System for Predicting the Recurrence of Colon Adenocarcinoma Patients

**DOI:** 10.3389/fonc.2020.00081

**Published:** 2020-02-06

**Authors:** Haojie Yang, Hong-Cheng Lin, Hua Liu, Dan Gan, Wei Jin, Can Cui, Yixin Yan, Yiming Qian, Changpeng Han, Zhenyi Wang

**Affiliations:** ^1^Department of Coloproctology, Yueyang Hospital of Integrated Traditional Chinese and Western Medicine, Shanghai University of Traditional Chinese Medicine, Shanghai, China; ^2^Department of Coloproctology, The Sixth Affiliated Hospital of Sun Yat-sen University (Gastrointestinal & Anal Hospital of Sun Yat-sen University), Guangzhou, China; ^3^Department of Emergency Medicine, Yueyang Hospital of Integrated Traditional Chinese and Western Medicine, Shanghai University of Traditional Chinese Medicine, Shanghai, China

**Keywords:** colon adenocarcinoma, differential expression analysis, risk score system, nomogram survival model, competitive endogenous RNA

## Abstract

Colon adenocarcinoma (COAD) is a common type of colon cancer, and post-operative recurrence and metastasis may occur in COAD patients. This study is designed to build a risk score system for COAD patients. The Cancer Genome Atlas (TCGA) dataset of COAD (the training set) was downloaded, and GSE17538 and GSE39582 (the validation sets) from Gene Expression Omnibus database were obtained. The differentially expressed RNAs (DERs) were analyzed by limma package. Using survival package, the independent prognosis-associated long non-coding RNAs (lncRNAs) were selected for constructing risk score system. After the independent clinical prognostic factors were screened out using survival package, a nomogram survival model was constructed using rms package. Furthermore, competitive endogenous RNA (ceRNA) regulatory network and enrichment analyses separately were performed using Cytoscape software and DAVID tool. Totally 404 DERs between recurrence and non-recurrence groups were identified. Based on the six independent prognosis-associated lncRNAs (including *H19, KCNJ2-AS1, LINC00899, LINC01503, PRKAG2-AS1*, and *SRRM2-AS1*), the risk score system was constructed. After the independent clinical prognostic factors (Pathologic M, pathologic T, and RS model status) were identified, the nomogram survival model was built. In the ceRNA regulatory network, there were three lncRNAs, four miRNAs, and 77 mRNAs. Additionally, PPAR signaling pathway and hedgehog signaling pathway were enriched for the mRNAs in the ceRNA regulatory network. The risk score system and the nomogram survival model might be used for predicting COAD recurrence. Besides, PPAR signaling pathway and hedgehog signaling pathway might affect the recurrence of COAD patients.

## Introduction

As a common malignancy occurring in the colon, colon cancer is divided into adenocarcinoma, undifferentiated carcinoma, and mucinous adenocarcinoma (1). Colon cancer is induced mainly by a high-fat low-fiber diet, and its symptoms include hematochezia, purulent stools, the change of bowel habits, abdominal mass, ileus, bellyache, and anemia (2). Colon cancer ranks third among the gastrointestinal cancers, and its morbidity and mortality rates in men are higher than those in women (3). The 5-years survival rate of colon cancer after radical operation is about 60–70%, and the main reasons leading to the failure of surgical treatment are post-operative recurrence and metastasis (4, 5). Therefore, the discovery of valuable molecular markers is important for the early prediction and treatment of colon cancer recurrence.

Long non-coding RNA (lncRNA) exerts a key regulatory effect in the development and progression of some diseases (6). In recent years, a variety of lncRNAs have been reported to be dysregulated in colon cancer. For example, the lncRNA small ubiquitin-like modifier 1 pseudogene 3 (*SUMO1P3*) is overexpressed in colon cancer, and is positively related to angiogenesis, metastases, advanced histological stages, and unfavorable outcome of colon cancer patients (7). Through activating the Wnt/β-catenin signaling, the lncRNA breast cancer anti-estrogen resistance 4 (*BCAR4*) accelerates the progression of colon cancer by promoting cell proliferation and suppressing cell apoptosis (8). The lncRNA cytoskeleton regulator RNA (*CYTOR*) facilitates epithelial-to-mesenchymal transition (EMT) and metastasis in colon cancer via interacting with β-catenin, which predicts poor prognosis and may be promising therapeutic target of colon cancer (9, 10). These results suggested that lncRNAs might be used as biomarkers for predicting the prognosis of colon cancer.

Along with the development of bioinformatics, several researchers have developed lncRNA-related signatures for predicting prognosis of colon cancer. Xue et al. proposed a two-lncRNA expression signature to predict survival of patients with colon adenocarcinoma (11). Xing et al. developed a 14-lncRNA prognostic signature for patients with colon adenocarcinoma (12). Lv et al. identified a five-lncRNA prognostic signature to predict the survival of colon cancer patients (13). These studies proved the strong power of prognostic prediction value of the lncRNA signatures for colon cancer patients. However, seldom studies were conducted to develop lncRNA signatures for predicting recurrence of colon cancer.

As a competitive endogenous RNA (ceRNA), lncRNA can mediate gene expression through acting as microRNA (miRNA) sponge (14, 15). The up-regulation of the lncRNA metastasis associated lung adenocarcinoma transcript 1 (*MALAT1*) mediates high mobility group box 1 (*HMGB1*) expression in colon cancer via competing with *miR-129-5p*, and the *MALAT1*/*miR-129-5p*/*HMGB1* axis serves as a key prognostic marker in the development of the tumor (16). In this study, the lncRNAs, miRNAs, and mRNAs with differential expression between recurrence and non-recurrence colon adenocarcinoma (COAD) samples were analyzed. The recurrence prognosis-associated lncRNAs were screened, and then the independent prognosis-associated lncRNAs were further selected for constructing risk score system. Moreover, nomogram survival model construction, ceRNA regulatory network construction and enrichment analysis were conducted. Our findings might be conducive to predicting the recurrence of COAD patients.

## Results

### Differential Expression Analysis

**Figure 1:**
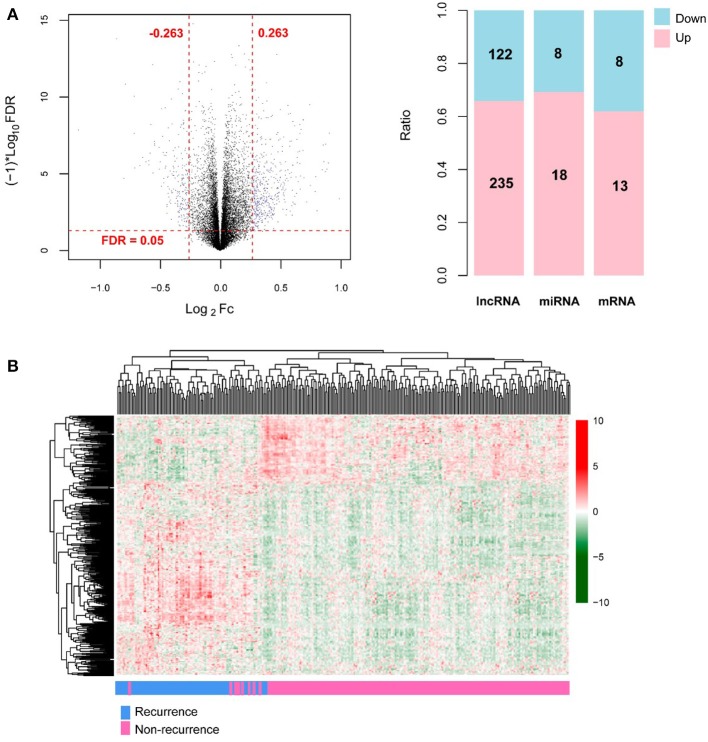
The screening results of differentially expressed RNAs (DERs). **(A)** The volcano plot (left; the horizontal dashed line represents false discovery rate (FDR) < 0.05, and the vertical dashed lines represent |log_2_ fold change (FC)| > 0.263; the blue dots indicate DERs; FC: fold change) and the histogram showing the proportional distribution of different kinds of DERs (right; pink and blue separately represent up-regulation and down-regulation; lncRNA: long non-coding RNA, miRNA: microRNA); **(B)** The hierarchical clustering heatmap (in sample strip, blue and pink separately represent recurrence samples and non-recurrence samples).

A total of 13,834 mRNAs, 827 lncRNAs, and 1,037 miRNAs were annotated from the TCGA transcriptomic RNA and miRNA datasets. The 310 COAD samples in the TCGA dataset were classified into recurrence (66 samples) and non-recurrence (244 samples) groups. Under the defined thresholds, 404 DERs (including 357 DE-mRNAs (122 down-regulated and 235 up-regulated), 26 DE-lncRNAs (eight down-regulated and 18 up-regulated), and 21 DE-miRNAs (eight down-regulated and 13 up-regulated) between recurrence and non-recurrence groups were screened out (Figure 1A). Based on the expression of the DERs, the clustering heatmap is drew and presented in Figure 1B.

### Construction of Risk Score System

Based on the univariable Cox regression analysis, a total of 21 DE-lncRNAs were found to be significantly related to recurrence prognosis (Table 1). From the 21 recurrence prognosis-associated lncRNAs, six independent prognosis-associated lncRNAs (including H19 imprinted maternally expressed transcript, *H19*; KCNJ2 antisense RNA 1, *KCNJ2-AS1*; long intergenic non-protein coding RNA 899, *LINC00899*; long intergenic non-protein coding RNA 1503, *LINC01503*; PRKAG2 antisense RNA 1, *PRKAG2-AS1*; and SRRM2 antisense RNA 1, *SRRM2-AS1*) were further identified (Table 2).

**Table 1:** Univariable Cox regression analysis identified 21 DE-lncRNAs related to recurrence prognosis.

**Symbol**	**coef**	**exp (coef)**	**se (coef)**	***z***	***P*-value**
*LINC01503*	0.834	2.3	0.209	4	6.40E-05
*LINC00899*	1.34	3.82	0.404	3.32	9.20E-04
*H19*	0.178	1.19	0.0547	3.26	1.10E-03
*LINC00894*	0.974	2.65	0.309	3.15	1.60E-03
*SRRM2-AS1*	3.37	29.2	1.13	2.98	2.90E-03
*HOXA-AS3*	0.55	1.73	0.187	2.94	3.30E-03
*C1RL-AS1*	0.905	2.47	0.317	2.86	4.20E-03
*PRKAG2-AS1*	−0.521	0.594	0.184	−2.82	4.70E-03
*HOXB-AS3*	0.309	1.36	0.11	2.81	5.00E-03
*SMG7-AS1*	2.18	8.87	0.843	2.59	9.60E-03
*PAX8-AS1*	0.338	1.4	0.136	2.49	1.30E-02
*BLACAT1*	0.338	1.4	0.138	2.44	1.50E-02
*LINC-PINT*	0.506	1.66	0.218	2.32	2.00E-02
*SLC2A1-AS1*	0.716	2.05	0.308	2.32	2.00E-02
*HOXB-AS1*	0.927	2.53	0.405	2.29	2.20E-02
*PTPRD-AS1*	−0.49	0.612	0.222	−2.21	2.70E-02
*LINC00589*	−2.06	0.127	0.948	−2.18	2.90E-02
*USP30-AS1*	−0.366	0.693	0.181	−2.03	4.20E-02
*LINC01116*	0.327	1.39	0.161	2.03	4.20E-02
*KCNJ2-AS1*	−0.874	0.417	0.435	−2.01	4.40E-02
*PRR7-AS1*	0.669	1.95	0.334	2	4.50E-02

**Table 2:** The information of the six independent prognosis-associated long non-coding RNAs (lncRNAs).

**Symbol**	**Coef**	***P*-value**	**Hazard ratio**	**95% CI**
*H19*	0.1647	2.23E-02	1.179	1.024–1.358
*KCNJ2-AS1*	−1.1438	1.60E-02	0.319	0.126–0.808
*LINC00899*	1.4922	1.65E-03	4.447	1.755–6.265
*LINC01503*	0.8525	1.22E-03	2.346	1.399–3.932
*PRKAG2-AS1*	−0.8100	6.73E-04	0.445	0.279–0.710
*SRRM2-AS1*	3.8523	1.35E-02	7.100	2.213–10.431

*CI, confidence interval*.

Subsequently, the risk score system based on the independent prognosis-associated lncRNAs was constructed, and the relevant formula was as follow:

$$\begin{array}{l} \text{RS}=\left(0.1647\right)\times \text{Ex}{\text{p}}_{\text{H}19}\;+\;\left(-1.1438\right)\times \text{Ex}{\text{p}}_{\text{KCNJ}2-\text{AS}1}\\ \;+\;\left(1.4922\right)\times \text{Ex}{\text{p}}_{\text{LINC}00899}\;+\;\left(0.8525\right)\times \text{Ex}{\text{p}}_{\text{LINC}01503}\\ \;+\;\left(-0.8100\right)\times \text{Ex}{\text{p}}_{\text{PRKAG}2-\text{AS}1}\;+\;\left(3.8523\right)\times \text{Ex}{\text{p}}_{\text{SRRM}2-\text{AS}1} \end{array}$$

**Figure 2:**
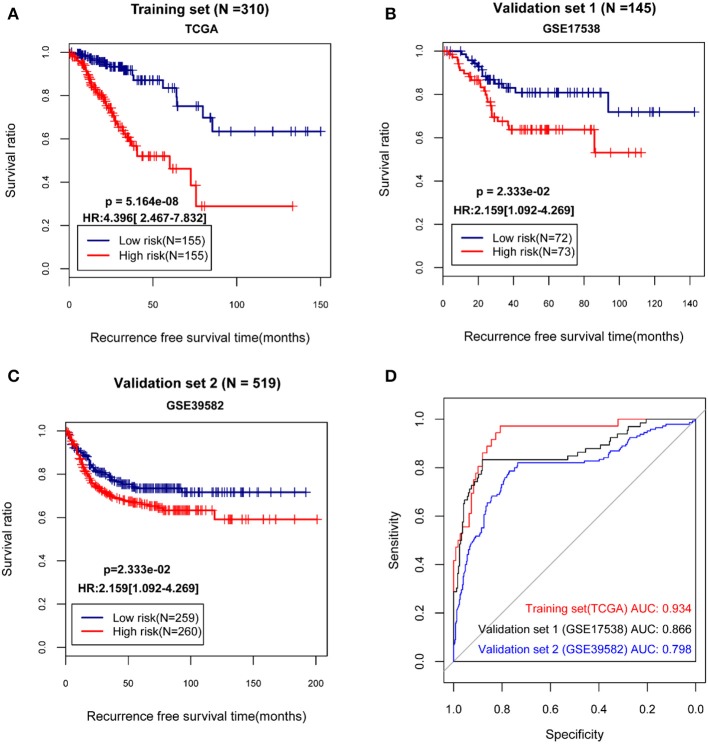
The Kaplan-Meier (KM) curves and receiver operating characteristic (ROC) curves showing the correlations between the grouping based on the risk score system and the actual recurrence prognosis. **(A)** The KM curves for The Cancer Genome Atlas (TCGA) dataset; **(B)** The KM curves for the validation set GSE17538; **(C)** The KM curves for the validation set GSE39582; **(D)** The ROC curves for the TCGA dataset and the validation sets; In KM curves, blue and red curves separately represent low risk group and high risk group. In ROC curves, red, black, and blue curves represent the TCGA dataset, the validation set GSE17538, and the validation set GSE39582, respectively. HR, hazard ratio; AUC, area under the receiver operating characteristic curve.

The median of the RSs of samples were calculated, and the samples in the TCGA dataset and the validation sets separately were divided into high and low risk group. Then, the correlation between the grouping and the actual recurrence prognosis information was evaluated using KM curves. The results showed that the grouping based on the risk score system had significant correlations with the actual recurrence prognosis in the TCGA dataset and the validation sets (Figure 2).

### Establishment and Validation of Nomogram Survival Model

**Figure 3:**
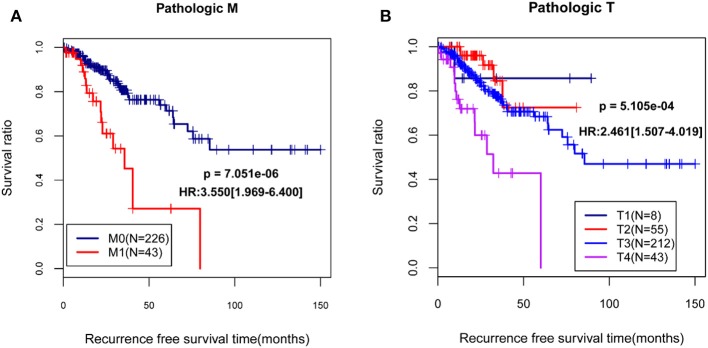
The Kaplan-Meier (KM) curves showed the associations between pathologic M/pathologic T and recurrence prognosis. **(A)** The KM curves for pathologic M (blue and red curves separately represent the samples in pathologic M0 stage and pathologic M1 stage); **(B)** The KM curves for pathologic T (black, red, blue, and purples curves represent the samples in pathologic T1 stage, pathologic T2 stage, pathologic T3 stage, and pathologic T4 stage, respectively). HR, hazard ratio.

By using univariable and multivariable Cox regression analyses, pathologic M, pathologic T, and RS model status were selected as the independent clinical prognostic factors in the COAD samples (Table 3). The COAD samples in lower pathologic M and pathologic T stages had better recurrence prognosis, which was consistent with clinical facts (Figure 3).

**Table 3:** The selection of independent clinical prognostic factors.

**Clinical characteristics**	**TCGA (*N* = 310)**	**Univariable cox**		**Multivariable cox**	
		**HR (95% CI)**	***P*-value**	**HR (95% CI)**	***P*-value**
Age (years, mean ± SD)	65.73 ± 12.71	0.994 [0.975–1.014]	5.549E-01	–	–
Gender (Male/Female)	169/141	1.802 [0.885–2.993]	2.103E-01	–	–
Pathologic M (M0/M1/–)	226/43/41	3.550 [1.969–6.400]	7.051E-06	3.450 [1.107–10.76]	3.280E-02
Pathologic N (N0/N1/N2)	180/77/53	1.948 [1.449–2.619]	4.638E-06	1.317 [0.749–2.314]	3.378E-01
Pathologic T (T1/T2/T3/T4)	8/55/212/35	2.461 [1.507–4.019]	5.105E-04	1.775 [1.167–3.259]	4.640E-02
Pathologic stage (I/II/III/IV/–)	51/118/88/43/10	1.761 [1.329–2.334]	6.207E-05	0.717 [0.334–1.542]	3.947E-01
Lymphatic invasion (Yes/No/–)	109/175/26	2.401 [0.441–3.997]	5.247E-02	–	–
Colon polyps history (Yes/No/–)	78/176/56	0.836 [0.443–1.578]	5.801E-01	–	–
RS model status (High/Low)	155/155	4.396 [2.467–7.832]	5.164E-08	3.793 [2.009–7.161]	3.940E-05
Recurrence (Yes/No)	66/244	–	–	–	–
Recurrence free survival time (months, mean ± SD)	29.66 ± 25.46	–	–	–	–

*TCGA, The Cancer Genome Atlas; HR, Hazard Ratio; CI, confidence interval*.

**Figure 4:**
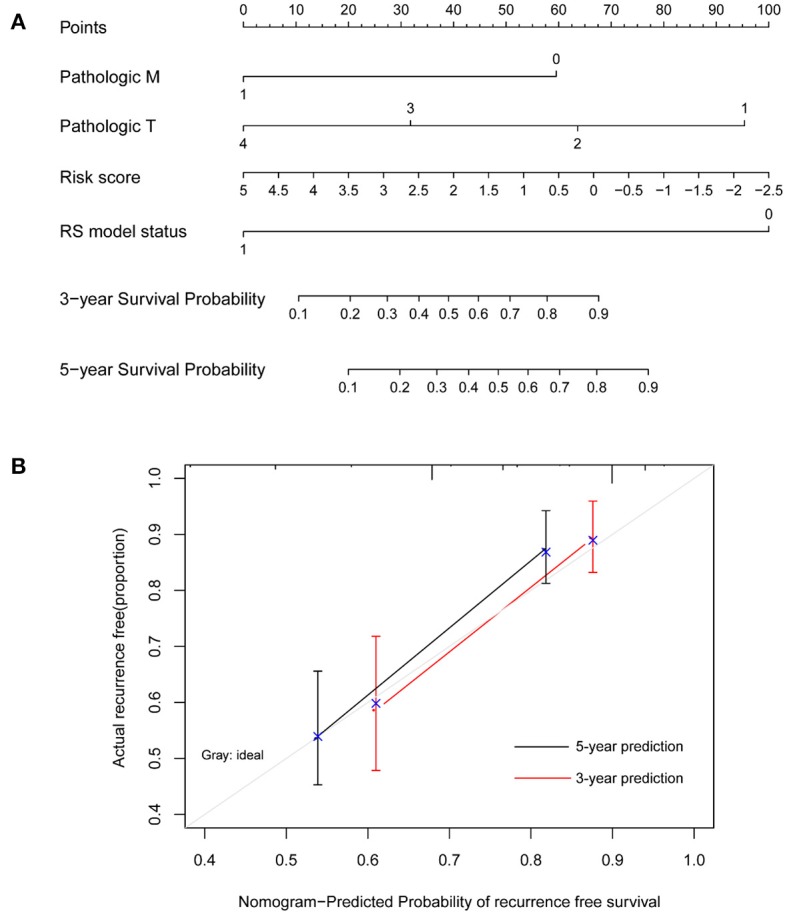
Nomogram survival model. **(A)** The nomogram survival model. **(B)** The graph showing the consistency of the nomogram-predicted survival probability and the actual survival probability (the horizontal and vertical axes represent the predicted survival probability and the actual survival probability, respectively; black and red segments separately represent the 5-years survival rate and the 3-years survival rate in the group with the highest consistency).

Moreover, a nomogram survival model was built based on the three independent prognostic factors (Figure 4A). The 3-years survival probability and 5-years survival probability of patients could be easily calculated based on their pathologic M, pathologic T and RS. Additionally, the nomogram-predicted 3-years survival probability/5-years survival probability was further compared with the actual 3-years survival probability/5-years survival probability recorded in TCGA and results suggested that there is high agreement between nomogram-predicted probability of recurrence free survival and the actual recurrence free survival (Figure 4B), indicating the good performance of nomogram survival model.

### CeRNA Regulatory Network Construction and Enrichment Analysis

**Figure 5:**
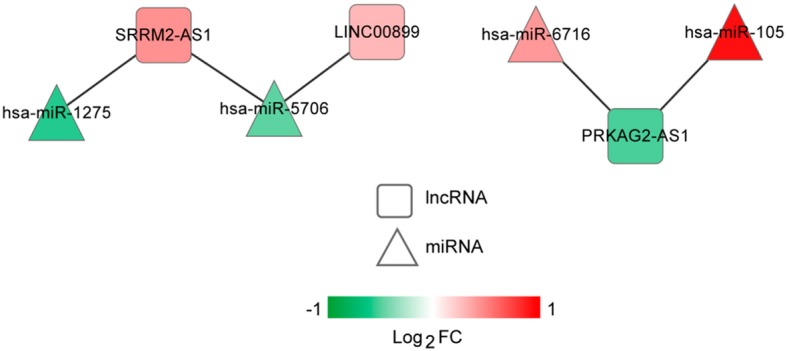
The long non-coding RNA (lncRNA)-microRNA (miRNA) regulatory network. Squares and triangles represent lncRNAs and miRNAs, respectively. Red and green separately represent up-regulation and down-regulation. FC, fold change.

**Figure 6:**
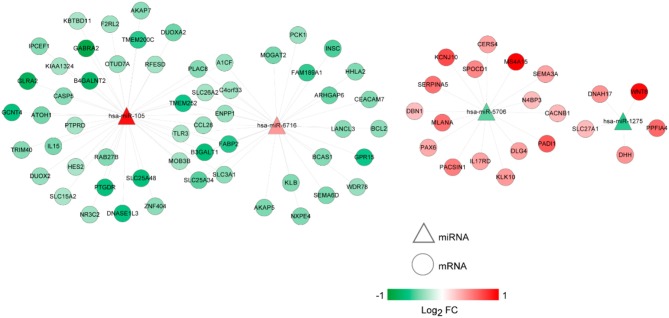
The microRNA (miRNA)-mRNA regulatory network. Circles and triangles represent mRNAs and miRNAs, respectively. Red and green separately represent up-regulation and down-regulation. FC, fold change.

**Figure 7:**
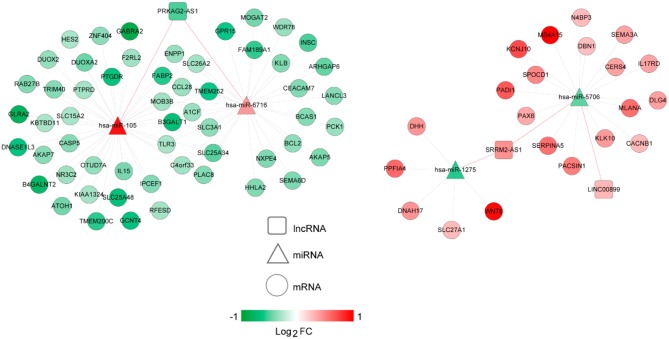
The competing endogenous RNA (ceRNA) regulatory network. Squares, circles, and triangles represent long non-coding RNAs (lncRNAs), mRNAs, and microRNAs (miRNAs), respectively. Red and green separately represent up-regulation and down-regulation. Red and black edges represent lncRNA-miRNA and miRNA-mRNA pairs, respectively. FC, fold change.

The lncRNA-miRNA regulatory network was constructed, which had seven nodes (three lncRNAs and four miRNAs) (Figure 5). Meanwhile, the miRNA-mRNA regulatory network was built, involving 81 nodes (four miRNAs and 77 mRNAs) (Figure 6). Based on the lncRNA-miRNA-mRNA relationships, the ceRNA regulatory network (84 nodes, including three lncRNAs (two up-regulated and one down-regulated), four miRNAs (two up-regulated and two down-regulated), and 77 mRNAs (21 up-regulated and 56 down-regulated) was constructed (Figure 7).

Based on DAVID online tool, 13 GO functional terms (such as regulation of neuron differentiation, *p*-value = 1.68E-03; regulation of neurogenesis, *p*-value = 3.74E-03; and inorganic anion transport, *p*-value = 5.45E-03) and five KEGG pathways (such as PPAR signaling pathway, *p*-value = 3.82E-03; neuroactive ligand-receptor interaction, *p*-value = 1.06E-02; intestinal immune network for IgA production, *p*-value = 2.00E-02; and hedgehog signaling pathway, *p*-value = 2.25E-02) were enriched for the mRNAs in the ceRNA regulatory network (Table 4).

**Table 4:** The enrichment results for the mRNAs in the competitive endogenous RNA (ceRNA) regulatory network.

**Category**	**Term**	**Count**	***P*-value**	**Genes**
GO_Biology process	GO:0045664~regulation of neuron differentiation	5	1.68E-03	*ATOH1, BCL2, PAX6, SEMA3A, DBN1*
	GO:0050767~regulation of neurogenesis	5	3.74E-03	*ATOH1, BCL2, PAX6, SEMA3A, DBN1*
	GO:0015698~inorganic anion transport	4	5.45E-03	*GABRA2, ENPP1, GLRA2, SLC26A2*
	GO:0051960~regulation of nervous system development	5	6.26E-03	*ATOH1, BCL2, PAX6, SEMA3A, DBN1*
	GO:0060284~regulation of cell development	5	7.86E-03	*ATOH1, BCL2, PAX6, SEMA3A, DBN1*
	GO:0006820~anion transport	4	1.75E-02	*GABRA2, ENPP1, GLRA2, SLC26A2*
	GO:0007015~actin filament organization	3	3.12E-02	*ARHGAP6, BCL2, DBN1*
	GO:0045017~glycerolipid biosynthetic process	3	3.79E-02	*MOGAT2, SEMA6D, PCK1*
	GO:0007409~axonogenesis	4	3.79E-02	*ATOH1, BCL2, PAX6, SEMA3A*
	GO:0048878~chemical homeostasis	6	4.61E-02	*DHH, ENPP1, BCL2, NR3C2, CCL28, PCK1*
	GO:0048667~cell morphogenesis involved in neuron differentiation	4	4.62E-02	*ATOH1, BCL2, PAX6, SEMA3A*
	GO:0048812~neuron projection morphogenesis	4	4.84E-02	*ATOH1, BCL2, PAX6, SEMA3A*
	GO:0030308~negative regulation of cell growth	3	4.87E-02	*ENPP1, BCL2, SEMA3A*
KEGG pathway	hsa03320:PPAR signaling pathway	3	3.82E-03	*SLC27A1, FABP2, PCK1*
	hsa04080:Neuroactive ligand-receptor interaction	4	1.06E-02	*F2RL2, GABRA2, PTGDR, GLRA2*
	hsa04672:Intestinal immune network for IgA production	2	2.00E-02	*IL15, CCL28*
	hsa04340:Hedgehog signaling pathway	2	2.25E-02	*DHH, WNT6*
	hsa04360:Axon guidance	2	4.47E-02	*SEMA6D, SEMA3A*

*GO, Gene Ontology; KEGG, Kyoto Encyclopedia of Genes and Genomes (KEGG)*.

## Discussion

In the present study, a total of 404 DERs (including 357 DE-mRNAs, 26 DE-lncRNAs, and 21 DE-miRNAs) between recurrence and non-recurrence groups were identified. After the 21 recurrence prognosis-associated lncRNAs were screened out, six independent prognosis-associated lncRNAs (including *H19, KCNJ2-AS1, LINC00899, LINC01503, PRKAG2-AS1*, and *SRRM2-AS1*) were further selected. Based on the independent prognosis-associated lncRNAs, the RS system was constructed. Pathologic M, pathologic T, and RS model status were identified as the independent clinical prognostic factors, and then the nomogram survival model was built. This nomogram survival model might be used to predict 3/5 years recurrence free survival in future clinical practice, by combining pathologic M, pathologic T and the RS calculated from the expression level of the 6 lncRNAs detected by qRT-PCR from surgical specimens.

High mobility group AT-hook 1 (*HMGA1*) is inhibited by *H19* short hairpin RNA (shRNA) and is promoted by *miR-138* inhibitor, and the *H19-miR138-HMGA1* pathway plays important roles in mediating the invasion and migration of colon cancer (17). *H19* expression is significantly up-regulated in immunodeficient mice induced by colon cancer cells, and *H19* may be taken as a novel therapeutic target in colon cancer (18). *H19* can suppress vitamin D receptor (*VDR*) expression via *miR-675-5p*, and increased *H19* leads to the resistance to 1,25(OH)2D3 treatment in the advanced colon cancer (19). *LINC00899* is elevated in the serum and bone marrow of acute myeloid leukemia (AML) patients, therefore, serum *LINC00899* may be a promising marker for the early diagnosis and prognosis of AML (20). These suggested that *H19* and *LINC00899* might be involved in the recurrence prognosis of COAD. Thirteen lncRNAs (including *KCNJ2-AS1*) are selected as prognostic biomarkers, based on which a prognostic signature is constructed for predicting the disease free survival in gastric cancer patients (21). Potassium voltage-gated channel subfamily J member 2 (*KCNJ2*)/*Kir2.1* belongs to the inwardly rectifying potassium channel family, which regulates drug resistance and cell growth through mediating mitochondrial 37S ribosomal protein MRP1 (*MRP1*)/ATP binding cassette subfamily C member 1 (*ABCC1*) expression in small-cell lung cancer (22). Up-regulated *LINC01503* contributes to cell proliferation, growth, invasion, and migration in esophageal squamous cell carcinoma (ESCC), which may be used as a marker of aggressive ESCC (23). Overexpressed *LINC01503* regulates forkhead box K1 (*FOXK1*) expression by competing with *miR-4492*, which promotes cell proliferation and invasion in colorectal cancer (24). Therefore, *KCNJ2-AS1* and *LINC01503* might also be correlated with the recurrence prognosis of COAD.

The genetic variation in a candidate pathway contributes to the risk of both colon and rectal cancers, and protein kinase AMP-activated non-catalytic subunit gamma 2 (*PRKAG2*), mechanistic target of rapamycin kinase (*FRAP1*), TSC complex subunit 2 (*TSC2*), and protein kinase AMP-activated catalytic subunit alpha 1 (*PRKAA1*) genes involved in the pathway are significantly related to colon cancer (25, 26). Through influencing the alternative splicing of downstream genes, the S346F mutation in serine/arginine repetitive matrix 2 (*SRRM2*) promotes the susceptibility of papillary thyroid carcinoma (27). Erb-b2 receptor tyrosine kinase 2 (*ERBB2*) overexpression is correlated with increased metastasis of human cancers, and the depletion of *SRRM2*, splicing factor, arginine/serine-rich 1 (*SFRS1*), *SFRS9*, and *SFRS10* proteins reduced the migration rate of ovarian cancer cells overexpressing *ERBB2* (28). These indicated that *PRKAG2-AS1* and *SRRM2-AS1* might play roles in the recurrence prognosis of COAD.

Through interacting with T cell transcription factor-4 (Tcf-4) and beta-catenin, peroxisome proliferator activated receptor gamma (PPAR gamma) may determine colon cell fate and serve as a target of the Wnt pathway in colon cancer cells (29). Hedgehog signaling pathway functions in gastrointestinal development and affects the formation of multiple tumors, which predicts unfavorable outcomes in colon cancer patients (30). The mRNAs in the ceRNA regulatory network were involved in PPAR signaling pathway and hedgehog signaling pathway, suggesting that PPAR signaling pathway and hedgehog signaling pathway might be related to the recurrence prognosis of COAD.

However, there are some limitations in this study. First, the differential expression of the 6 lncRNAs was identified from RNA sequence data. Experiment validation of these lncRNAs in colon cancer patients should be conducted in further research. Second, the ceRNAs related with these lncRNAs should also be validated in *in vitro* and *in vivo* studies.

In conclusion, 357 DE-mRNAs, 26 DE-lncRNAs, and 21 DE-miRNAs were identified between recurrence and non-recurrence groups. Besides, the risk score system (involving *H19, KCNJ2-AS1, LINC00899, LINC01503, PRKAG2-AS1*, and *SRRM2-AS1*) and the nomogram survival model might be useful for the prediction of COAD recurrence. Moreover, PPAR signaling pathway and hedgehog signaling pathway might be correlated with the recurrence prognosis of COAD patients.

## Materials and Methods

### Data Source

From The Cancer Genome Atlas (TCGA) database (https://cancergenome.nih.gov/), the transcriptomic RNA (including 512 samples) and miRNA (including 461 samples) expression data of COAD (downloaded in May 15, 2019; platform: Illumina HiSeq 2000 RNA Sequencing) was extracted. The two groups of samples were paired according to the sample numbers, and then were corresponded to the downloaded clinical information. Finally, a total of 310 paired COAD samples with recurrence prognosis information were screened and used as the training set.

Taking “colon adenocarcinoma” and “Homo sapiens” as searching words, the eligible datasets were selected from Gene Expression Omnibus (GEO) database (http://www.ncbi.nlm.nih.gov/geo/) according to the following criteria: (1) the samples were solid tissue samples from COAD patients; (2) the total sample size was no <200, and the number of COAD samples was not under 150; (3) the COAD samples had recurrence prognosis information. After searching, two datasets (GSE17538, 244 samples (including 145 COAD samples with recurrence prognosis information), platform: GPL570 Affymetrix Human Genome U133 Plus 2.0 Array, the validation set 1; and GSE39582, 585 samples (including 519 COAD samples with recurrence prognosis information), platform: GPL570 Affymetrix Human Genome U133 Plus 2.0 Array, the validation set 2) that met the requirements were downloaded.

### Differential Expression Analysis

Using HUGO Gene Nomenclature Committee (HGNC) database (http://www.genenames.org/) (31), the lncRNAs and mRNAs in the datasets were recognized. Based on the source information, the samples in the TCGA dataset were classified into recurrence and non-recurrence groups. The R package limma (version 3.34.7, https://bioconductor.org/packages/release/bioc/html/limma.html) (32) was applied for exploring the differentially expressed RNAs [DERs, including differentially expressed lncRNAs (DE-lncRNAs), differentially expressed mRNAs (DE-mRNAs), and differentially expressed miRNAs (DE-miRNAs)] between recurrence and non-recurrence groups. The false discovery rate (FDR) < 0.05 and |log_2_ fold change (FC)| > 0.263 were taken as the screening criteria. Using the R package pheatmap (version 1.0.8, https://cran.r-project.org/web/packages/pheatmap/index.html) (33), the expression values of the DERs in TCGA dataset were conducted with bidirectional hierarchical clustering.

### Construction of Risk Score System

Based on the recurrence information of the 310 COAD samples in the TCGA dataset, the DE-lncRNAs having significant associations with recurrence prognosis were selected combined with the univariable Cox regression analysis in the survival package (version 2.41-1, http://bioconductor.org/packages/survivalr/) in R (34). The threshold was set as log-rank *p*-value < 0.05.

From the recurrence prognosis-associated lncRNAs, the independent prognosis-associated lncRNAs were further selected using the multivariable Cox regression analysis in the survival package in R (34). Afterwards, the risk score system was built based on the expression levels and independent prognostic coefficients of the independent prognosis-associated lncRNAs. The risk scores (RSs) of the COAD samples were calculated using the formula below:

$$\begin{array}{l} \text{Risk score }\left(\text{RS}\right)\;=\;\sum \beta_{\text{lncRNA}}\times \text{Ex}{\text{p}}_{\text{lncRNA}} \end{array}$$

β _lncRNA_ indicates the independent prognostic coefficient of independent prognosis-associated lncRNA, and Exp_lncRNA_ stands for the expression level of independent prognosis-associated lncRNA.

The median of the RSs of the COAD samples in the TCGA dataset were calculated and used to divide the samples into high risk group and low risk group. Using the Kaplan-Meier (KM) curve in the R package survival (34), the association between the grouping and the actual recurrence prognosis information was analyzed. Similarly, the prediction efficiencies of the risk score system in the validation sets were evaluated.

### Construction of Nomogram Survival Model

Using the univariable and multivariable Cox regression analyses in the R package survival (34), the independent clinical prognostic factors in the COAD samples in the TCGA dataset were identified. The log-rank *p*-value < 0.05 was selected as the significant threshold. Using the R package rms (version 5.1-2, https://cran.r-project.org/web/packages/rms/index.html) (35), nomogram survival model was constructed based on the independent clinical prognostic factors and the predicted risk information.

### CeRNA Regulatory Network Analysis and Enrichment Analysis

The DIANA-LncBase v2 database (http://carolina.imis.athena-innovation.gr/diana_tools/web/index.php?r=lncbasev2%2Findex-experimental) (36) was used to search the relationships between the DE-miRNAs and the independent prognosis-associated lncRNAs. The pairs involving miRNAs and lncRNAs with opposite expression directions were selected, based on which the lncRNA-miRNA regulatory network was visualized using Cytoscape software [version 3.6.1, https://cytoscape.org/ (37)].

The starBase database (version 2.0, http://starbase.sysu.edu.cn/, including the information of RNA22, PITA, targetScan, picTar, and miRanda databases) (38) was applied for searching the targets of the miRNAs implicated in the lncRNA-miRNA regulatory network. The regulatory relationships included by at least two of RNA22, PITA, targetScan, picTar, and miRanda databases were taken as the miRNA-target pairs. Subsequently, the pairs involving negatively correlated miRNAs and DE-mRNAs were further screened. Moreover, Cytoscape software (37) was utilized to visualize the miRNA-mRNA regulatory network.

The lncRNA-miRNA-mRNA regulatory relationships were obtained through integrating the lncRNA-miRNA and miRNA-mRNA pairs. Based on the lncRNA-miRNA-mRNA regulatory relationships, the ceRNA regulatory network was built by Cytoscape software (37). Using DAVID online tool (version 6.8, https://david.ncifcrf.gov/) (39), Kyoto Encyclopedia of Genes and Genomes (KEGG) pathway and Gene Ontology (GO) functional enrichment analyses for the mRNAs in the ceRNA regulatory network were carried out. The *p*-value < 0.05 was selected as the threshold of enrichment significance.

## Data Availability Statement

The datasets generated for this study can be found in The Cancer Genome Atlas (https://portal.gdc.cancer.gov/); the NCBI Gene Expression Omnibus (GSE17538, GSE39582).

## Author Contributions

CH and ZW: funding acquisition. HY, CH, and ZW: investigation. HY, H-CL, HL, DG, YQ, CH, and ZW: methodology. H-CL, CH, and ZW: project administration. HY, DG, WJ, CC, and YY: software. HL: supervision. H-CL: validation. HY, HL, CH, and ZW: writing–original draft.

### Conflict of Interest

The authors declare that the research was conducted in the absence of any commercial or financial relationships that could be construed as a potential conflict of interest.
